# Therapeutic effects of a synthetic glabridin derivative on metabolic immuno-regulation and salivary gland functional restoration in experimental Sjögren’s syndrome

**DOI:** 10.1038/s12276-026-01778-0

**Published:** 2026-07-21

**Authors:** Jin-Sil Park, Hye Yeon Kang, Ha Yeon Jeong, JeongWon Choi, Sang Hee Cho, Su Beom Lee, Mi-La Cho, Sung-Hwan Park

**Affiliations:** 1 Rheumatism Research Center, Catholic Research Institute of Medical Science, College of Medicine, The Catholic University of Korea, Seoul, 06591 South Korea; 2 Lab of Translational ImmunoMedicine, Catholic Research Institute of Medical Science, College of Medicine, The Catholic University of Korea, Seoul, 06591 South Korea; 3 Department of Pathology, College of Medicine, The Catholic University of Korea, Seoul, 06591 South Korea; 4 Mitochondrial Immunomedicine Center for Global Health Therapeutics, Catholic Research Institute of Medical Science, College of Medicine, The Catholic University of Korea, Seoul, 06591, South Korea; 5 Department of Medical Sciences, Graduate School of The Catholic University of Korea, Seoul, 06591 South Korea; 6 Divisions of Rheumatology, Department of Internal Medicine, Seoul St. Mary’s Hospital, College of Medicine, The Catholic University of Korea, Seoul, 06591, South Korea

**Keywords:** Autoimmunity, Autoimmune diseases, Chronic inflammation, Gene regulation in immune cells

## Abstract

Sjögren’s syndrome (SS) is a chronic autoimmune disease in which inflammatory cells infiltrate the exocrine glands, reducing glandular secretory function and ultimately resulting in keratoconjunctivitis sicca (dry eyes) and xerostomia (oral dryness). Cardiovascular risk factors are more prevalent in patients with SS than in healthy controls; patients with SS and metabolic syndrome also have higher leptin and inflammatory cytokine levels. In this study, we investigated the effects of HGR4113, a structural analogue of glabridin that promotes mitochondrial function and is in clinical trials for obesity treatment, on the development of SS in non-obese diabetic NOD/ShiLtJ mice. HGR4113 inhibited IL-17 production by regulating STAT3 activity and the metabolic profile of splenic CD4^+^ T cells; it also increased the frequency of IL-10-producing regulatory B cells and decreased immunoglobulin production. Oral administration of HGR4113 (100 mg/kg) improved salivary flow rate, reduced lymphocyte infiltration and lowered inflammatory cytokine levels in the salivary glands of NOD/ShiLtJ mice. HGR4113 also decreased the frequencies of splenic IL-17-producing T and B cells, germinal-centre B cells and plasma cells ex vivo in NOD/ShiLtJ mice. Additionally, 3D epithelial structure formation from salivary gland cells of HGR4113-treated NOD/ShiLtJ mice increased, as did the levels of E-cadherin, aquaporin-5, α-SMA and cytokeratin-14. Finally, treatment with HGR4113 promoted the development of 3D salivary epithelial structures in vitro. HGR4113 improves salivary gland hypofunction by inhibiting lymphocyte infiltration and inflammation in the salivary glands and restoring damaged salivary tissue in SS-like NOD/ShiLtJ mice.

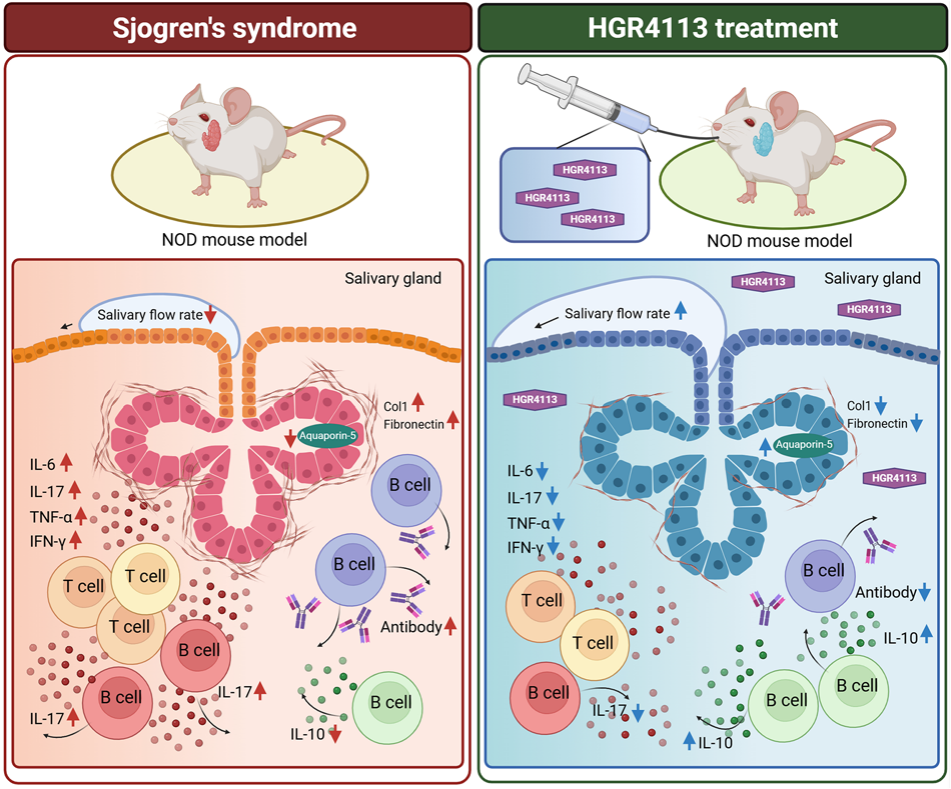

## HGR4113 Enhances Salivary Function in Mice with Sjögren’s Syndrome

Sjögren’s syndrome (SS) is a chronic autoimmune disease characterized by inflammation and reduced glandular function, often leading to severe complications. This study explores the potential of HGR4113, a novel glabridin analogue, in modulating immune responses and alleviating SS symptoms in a mouse model. Researchers administered HGR4113 to NOD/ShiLtJ mice and assessed its effects on immune-cell activity and salivary-gland function. HGR4113 significantly reduced inflammatory cytokine production and Th17 cell differentiation by inhibiting STAT3 phosphorylation, while promoting regulatory B cell development. It also improved salivary gland function, reducing fibrosis and enhancing epithelial structure formation. These findings suggest HGR4113 as a promising candidate for SS treatment, offering a targeted approach by modulating mitochondrial metabolism and immune responses. Future research could further explore its long-term regenerative potential and clinical applicability in SS patients.

This summary was initially drafted using artificial intelligence, then revised and fact-checked by the author.

## Introduction

Sjögren’s syndrome (SS) is a chronic autoimmune disease in which inflammatory cells infiltrate the exocrine glands, reducing glandular secretory function and ultimately resulting in keratoconjunctivitis sicca (dry eyes) and xerostomia (oral dryness). SS is often accompanied by extraglandular manifestations involving the skin, muscles, kidneys, joints, peripheral nervous system and blood vessels^1,2^. In SS, mortality is increased in patients with extraglandular manifestations and non-Hodgkin lymphoma^2^.

The pathogenesis of SS is still under investigation, but lymphocyte infiltration into target organs, including exocrine glands such as the salivary and lacrimal glands, is considered a key mechanism of disease development^3^. Aggregates of B and T cells are observed in ectopic lymphoid structures in the salivary and lacrimal glands, and B cells that receive signals from T cells become hyperactivated, resulting in hypergammaglobulinaemia, germinal-centre (GC) formation, and production of anti-SSA/Ro and anti-SSB/La autoantibodies^4,5^. Metabolic imbalance can alter immune-cell phenotypes and immune responses, potentially leading to autoimmunity or malignant transformation^6,7^. Recently, metabolic abnormalities have been reported to play a role in the pathogenesis of SS. Ultrastructural alterations of mitochondria have been observed in the salivary epithelial cells of patients with SS^8^, and oxidative stress levels are increased in their saliva^9^.

Glabridin, a polyphenolic isoflavan isolated from the root of *Glycyrrhiza glabra* L. (Fabaceae), commonly known as liquorice, is a widely used herb-derived natural compound with anti-inflammatory, antioxidant, anticancer and antiatherogenic effects, as well as the ability to regulate energy metabolism^10,11^. However, glabridin has poor stability in formulations and low bioavailability, limiting its application as a commercial clinical therapeutic agent^12,13^. A recent structure–activity relationship study identified a novel structural analogue of glabridin, HGR4113 ((R)-2-(8,8-dimethyl-2,3,4,8,9,10-hexahydropyrano[2,3-f]chromen-3-yl)-5-propoxyphenol), with improved chemical stability and metabolic characteristics relative to glabridin^14^.

In the present study, we evaluated the ability of HGR4113 to modulate inflammatory responses in murine splenic T and B cells and its therapeutic efficacy in NOD/ShiLtJ mice, a preclinical model of SS. We also investigated the role of HGR4113 in 3D salivary epithelial structure formation in NOD/ShiLtJ mice ex vivo and in vitro.

## Materials and methods

### Animals

Seven-week-old female NOD/ShiLtJ mice were purchased from The Jackson Laboratory (Bar Harbor, ME, USA) and the cage of the animals was maintained under specific pathogen-free conditions at the Institute of Medical Science, Catholic University of Korea. The mice were fed standard mouse chow and water. All procedures were approved by the Animal Research Ethics Committee of the Catholic University of Korea and conformed to the United States National Institutes of Health guidelines (permit number: CUMS-2024-0267-02). All animals were treated and euthanized in accordance with the Guidelines on the Use and Care of Animals of the Catholic University of Korea. Surgery was performed under isoflurane anaesthesia, and every effort was made to minimize suffering. At the end of the study, the mice were euthanized in a CO_2_ chamber for sample collection.

### Treatment with HGR4113 and measurement of saliva secretion in NOD/ShiLtJ mice

Saliva secretion was stimulated in anaesthetized mice by intraperitoneal injection of pilocarpine (Sigma-Aldrich, St. Louis, MO, USA) at a dose of 5 mg/kg body weight. Saliva was collected from the oral cavity for 7 min, beginning 90 s after the injection, using a micropipette. The volume of saliva was determined gravimetrically (μl/g/min). Before drug injection, salivary flow rates were measured in mice, and groups were randomized based on the average salivary flow rate of all mice. HGR4113 was dissolved in dimethyl sulfoxide. Seven-week-old female NOD/ShiLtJ mice were administered 100 mg/kg of HGR4113 or vehicle orally once daily for 12 weeks (*n* = 10/group). Longitudinal monitoring of blood glucose confirmed that no individuals reached the exclusion threshold of 600 mg/dl; thus, no mice were excluded owing to diabetes development during the study. To ensure robust assessment of long-term safety, body weight was recorded weekly throughout the 12-week treatment period (Supplementary Fig. 1a). The 100-mg/kg dose was selected based on the Human Equivalent Dose conversion of the clinical dose range (NCT05642377). This selection was further validated by a preliminary dose-finding study comparing 100 mg/kg and 200 mg/kg (Supplementary Fig. 2) and by prior pharmacokinetic data indicating that doses within the 80–160 mg/kg range achieve peak plasma concentration (*C*_max_) levels consistent with the effective concentrations used in our in vitro assays^14^. Sample size was determined for statistical significance and experimental feasibility. To minimize potential confounders such as the order of treatments and measurements, we individually marked each mouse with an ear punch. Blood-glucose levels were measured using an Accu-Check Compact Glucometer (Roche Diagnostics, Indianapolis, IN, USA). Individuals with blood-glucose levels of 600 mg/dl were excluded from evaluation owing to the development of diabetes. Samples were stored at −70°C until analysis.

### Histopathological examination and immunohistochemistry

Salivary glands and spleen tissues were fixed in 4% paraformaldehyde and embedded in paraffin. Spleen tissues were specifically processed for haematoxylin and eosin staining to evaluate the potential systemic and lymphoid organ toxicity of HGR4113. Five-micrometre-thick sections were stained with haematoxylin and eosin, and the degree of inflammation in the salivary gland was quantified as previously described^15^. Additionally, analysis of splenic histology focused on maintaining the structural integrity of white and red pulp and assessing any treatment-related pathological alterations (Supplementary Fig. 1b). Sections were incubated with primary antibodies against CD4 (1:50, Abcam, #ab25475), CD19 (1:200, Abcam, #ab203615), IL-6 (1:300, Novus, #NB600-1131), IL-17 (1:200, Abcam, #ab79056), type I collagen (Col-I) (1:100, Invitrogen, #PA5-89281), fibronectin (1:200, Abcam, #ab2413), TGF-β (1:100, Bioss, #BS-0086R), and aquaporin-5 (AQP5) (1:100, Novus, #NBP2-92926) for 2 h at room temperature. Sections were then incubated with a horseradish peroxidase-conjugated secondary antibody for 30 min. The final products were visualized using the chromogen diaminobenzidine. Immunostained sections were examined by photomicroscopy (Olympus). To ensure objectivity, all histological and immunofluorescent analyses were performed in a blinded manner. Positive cells were counted in high-power digital images (×400 magnification) by three independent investigators who were blinded to the experimental groups.

### Isolation and stimulation of splenocytes

Murine splenocytes were prepared as previously described^16^. Spleens were dissociated using sterilized glass slides with frosted ends, and red blood cells were lysed in hypotonic ACK buffer (0.15 mM NH_4_Cl, 1 mM KCO_3_, and 0.1 mM ethylenediaminetetraacetic acid, pH 7.4). The remaining splenocytes were filtered through a 40-µm cell strainer (Falcon, Durham, NC, USA) and maintained in RPMI 1640 medium containing 5% fetal bovine serum (FBS; Thermo Fisher Scientific, MA, USA). Splenic CD4^+^ T or CD19^+^ B cells were purified using a positive cell isolation kit according to the manufacturer’s instructions (Miltenyi Biotec). The cells were pretreated with HGR4113 for 2 h. Splenocytes or isolated T cells were cultured with 0.5 µg/ml anti-CD3 (BD Pharmingen, #553057) and 1 µg/ml anti-CD28 (BD Pharmingen, #553294) antibodies, or under Th17-polarizing conditions with anti-CD3, anti-CD28, 20 ng/ml IL-6 (R&D Systems, #406-ML), 2 ng/ml transforming growth factor-β (TGF-β) (PeproTech, #100-21 C), 5 µg/ml anti-IFNγ antibody (R&D Systems, #MAB485) and 5 µg/ml anti-IL-4 antibody (R&D Systems, #MAB404) for 72 h. Splenocytes or isolated B cells were cultured with 100 ng/ml lipopolysaccharide (LPS) (Sigma-Aldrich, #L4391) or 10 µg/ml IgM antibody (Jackson ImmunoResearch Labs), 20 ng/ml recombinant mouse IL-4 protein (R&D Systems) or 1 µg/ml recombinant murine sCD40 ligand (PeproTech) for 72 h.

### Seahorse metabolic assay

The oxygen consumption rate (OCR) was measured using an XF96 Extracellular Flux Analyzer (Seahorse Bioscience) according to the manufacturer’s instructions. Briefly, 5 × 10^5^ purified splenic CD4^+^ T cells were cultured in RPMI 1640 medium with 5% FBS in the presence or absence of HGR4113 under anti-CD3 and anti-CD28 antibodies stimulation for 12 h. The washed cells, resuspended in XF medium containing 1× ITSA (Gibco), were seeded on Cell-Tak–coated (BD Biosciences) Seahorse cell-culture plates and incubated in an incubator at 37°C without CO_2_ for 30 min. Real-time changes in OCR (pmol O_2_/min) were measured after treatment with oligomycin (complex V inhibitor, 7 μM), FCCP (3 μM), and rotenone plus antimycin A (electron transport chain inhibitors, 10 μM).

### Real-time polymerase chain reaction

Total RNA was extracted using TRI Reagent (Molecular Research Center), and cDNA was synthesized with the Dyne First-Strand cDNA Synthesis Kit (Dyne Bio) according to the manufacturer’s protocol. Gene expression was measured using the StepOnePlus Real-Time PCR System (Applied Biosystems) with SYBR Premix (Bioline Inc.). The following primers were used: *interferon* (*Ifn)g*, 5′-AGG AAC TGG CAA AAG GAT GGT-3′ and 5′-ATG TTG TTG CTG ATG GCC TG-3′; *interleukin* (*IL)-17*, 5′-CCT CAA AGC TCA GCG TGT CC-3′ (sense) and 5′-GAG CTC ACT TTT GCG CCA AG-3′ (antisense); *Foxp3*, 5′-CAC TGC CCC TAG TCA TGG T-3′ (sense) and 5′-GGA GGA GTG CCT GTA AGT GG-3′ (antisense); *IL-10*, 5′-GGC CCA GAA ATC AAG GAG CA-3′ (sense) and 5′-AGA AAT CGA TGA CAG CGC CT-3′ (antisense); *BAFFR*, 5′-GGT GGG TCT AGT GAG TCT GGT-3′ (sense) and 5′-TTC TGA GGA GGG TAC AAA GAC AT-3′ (antisense); activation-induced cytidine deaminase (*AICDA*), 5′-GCC ACC TTC GCA ACA AGT CT-3′ (sense) and 5′-CCG GGC ACA GTC ATA GCA C-3′ (antisense); *Col1a1*, 5′-GCC AAG AAG ACA TCC CTG AAG-3′ (sense) and 5′-TGT GGC AGA TAC AGA TCA AGC-3′ (antisense); and *β-actin*, 5′-GTA CGA CCA GAG GCA TAC AGG-3′ (sense) and 5′-GAT GAC GAT ATC GCT GCG CTG-3′ (antisense). mRNA levels were normalized to those of *β-actin*.

### Immunoblot analysis

Cells were lysed in Halt protein lysis buffer containing Halt phosphatase inhibitor (Thermo Pierce). Lysates were centrifuged at 14,000 × *g* for 15 min at 4°C. Protein concentration was determined using the Bradford protein assay (Bio-Rad). Protein samples were separated by 10% sodium dodecyl sulfate–polyacrylamide gel electrophoresis and transferred to Hybond membranes (Amersham Pharmacia Biotech, Piscataway, NJ, USA). Membranes were incubated with antibodies against phosphorylated STAT3 (Y705, Abcam, #ab76315), STAT3 (Abcam, #ab68153) and glyceraldehyde-3-phosphate dehydrogenase (GAPDH; Abcam, #ab181602). Hybridized bands were detected using an ECL detection kit (Pierce) and Hyperfilm (Agfa). Western blot analysis was performed using the SNAP i.d. Protein Detection System (Millipore). Protein levels were normalized to β-actin or GAPDH as loading controls, and the relative level of the protein of interest under the nil condition was set to 1.

### Enzyme-linked immunosorbent assay

Tumour necrosis factor (TNF)-α, IFNγ, IL-17 and IL-6 levels in culture supernatants were determined by sandwich ELISA (DuoSet; R&D Systems, Lille, France). Blood was collected from the orbital sinuses of mice, and serum samples were stored at −20°C until use. Serum IgG concentration was measured using the ELISA Quantitation Set (Bethyl Laboratories) according to the manufacturer’s protocol. Streptavidin-conjugated horseradish peroxidase was used for colour development. Absorbance at 450 nm was measured using an ELISA microplate reader (Molecular Devices, Sunnyvale, CA, USA). To ensure reproducibility, serum samples from independent experimental batches were integrated for total IgG analysis (*n* = 15 for vehicle, *n* = 9 for HGR4113).

### Intracellular staining and flow cytometry

To stain surface markers, single-cell suspensions were washed with fluorescence-activated cell sorting (FACS) buffer (phosphate-buffered saline [PBS] with 2% FBS) and incubated with fluorochrome-labelled antibodies for 30 min at 4°C. For intracellular staining, single-cell suspensions were cultured with 25 ng/ml phorbol myristate acetate (PMA; Sigma-Aldrich) and 250 ng/ml ionomycin (Sigma-Aldrich) with added GolgiStop (BD Biosciences) for 4 h. After surface staining, the cells were fixed and permeabilized with Cytofix/Cytoperm according to the manufacturer’s instructions (BD Biosciences). For intracellular Foxp3 and Bcl-6 staining, a Foxp3/Transcription Factor Staining Buffer Kit (eBioscience) was used following surface staining. After washing with Perm/Wash buffer, antibodies for intracellular staining were added for 30 min at 4 °C. The following anti-mouse antibodies were used for FACS: Alexa Fluor® 700 anti-CD4 (RM4-5), PE-Cy7 anti-CD19 (1D3), PerCP anti-CD45R/B220 (RA3-6B2), PE anti-CD138 (281-2) and FITC anti-GL-7 (GL7) from BD Biosciences; PerCP-Cy5.5 anti-CD4 (RM4-5), eFluor 450 anti-CD4 (RM4-5), FITC anti-CD5 (53-7.3), PE anti-CD1d (1B1), PE anti-Fas (15A7), PerCP-eFluor 710 anti-CXCR5 (SPRCL5), PE-Cyanine7 anti-ICOS (7E.17G9), APC anti-Bcl-6 (BCL-DWN), PE anti-Foxp3 (FJK-16s), PE anti-IFNγ (XMG1.2), PE anti-IL-17 (eBio17B7) and APC anti-IL-10 (JES5-16E3) from Invitrogen; and APC anti-CD25 (PC61) from BioLegend. Stained cells were analysed on a FACSCalibur (BD Biosciences), CytoFLEX (Beckman Coulter) or LSRII (BD Biosciences). Events were collected and analysed using FlowJo software (Tree Star). The final flow cytometry analysis included the full cohort (*n* = 10 per group) to ensure statistical power.

### Mouse salivary gland 3D epithelial primary culture

Spheroid-forming mouse salivary gland stem cells were isolated from the submandibular gland. The thin fascia covering the submandibular gland was carefully removed to obtain the tissue. The tissue was washed three times with PBS (Gibco) containing 3% penicillin–streptomycin and minced into small pieces, which were placed in Dulbecco’s Modified Eagle’s Medium (DMEM; Gibco) containing 1 mg/ml collagenase IV (Gibco) and incubated for 30 min at 37°C in a 5% CO_2_ atmosphere. The tissue was then centrifuged, and the cell pellet was resuspended in DMEM and filtered through a 40-µm cell strainer (BD) to obtain single cells. After centrifugation, the cells were cultured in DMEM/F12 (1:1, v/v; Gibco) supplemented with 20 ng/ml epidermal growth factor (PeproTech), 20 ng/ml fibroblast growth factor-2 (PeproTech), 1% N2 supplement (Gibco), 1% insulin–transferrin–selenium (Gibco), 1 μM dexamethasone (Sigma) and 1% antibiotic–antimycotic (GenDEPOT). Salivary gland stem cells were seeded on Matrigel (Corning) and cultured for 10 days to develop 3D epithelial structures. For 3D culture assays, *n* = 5 mice from the vehicle group were selected based on their salivary flow rates being closest to the group mean to ensure biological representativeness. For the HGR4113 group, *n* = 9 samples were initially processed, with final imaging performed on *n* = 8 samples owing to sample exhaustion during multiple staining rounds.

### Immunofluorescence

Salivary gland 3D epithelial structures were snap-frozen in liquid nitrogen and stored at −70°C. Six-micrometre-thick cryosections were fixed in acetone for 10 min and dried at room temperature. The slides were hydrated in Tris buffer and blocked with 10% goat serum for 30 min at room temperature. They were then incubated overnight at 4°C with the appropriate antibodies diluted in Tris buffer. Sections were mounted with fluorescence mounting medium (Dako). The following anti-mouse antibodies were used for staining: keratin (KRT) 5 (1:100, Abcam, #ab52635), KRT7 (1:200, Invitrogen, #MA1-06315), α-SMA (1:800, Abcam, #ab7817), KRT14 (1:600, Abcam, #ab181595), E-cadherin (1:1000, Abcam, #ab231303) and AQP5 (1:100, Novus, #NBP2-92926). Nuclei were stained with DAPI (D35271; Invitrogen) in PBS. All stained sections were imaged using a confocal laser scanning microscope (LSM700).

### Statistical analysis

Statistical analyses were performed using GraphPad Prism (version 9 for Windows; GraphPad Software, San Diego, CA, USA). The normality of the data distribution was assessed using the Shapiro–Wilk test, and the homogeneity of variances was verified using Levene’s test to ensure that parametric assumptions were met. Normally distributed continuous data were analysed using the parametric Student’s *t* test for comparisons between two groups. For comparisons involving more than two groups, differences in mean values were analysed using one-way analysis of variance (ANOVA), followed by Tukey’s honestly significant difference (HSD) post hoc test for multiple comparisons. Data are presented as mean ± standard deviations (SD). *P* values < 0.05 (two-tailed) were considered statistically significant.

## Results

### HGR4113 reduces the production of inflammatory cytokines in splenocytes from SS-like NOD/ShiLtJ mice

**Fig. 1:**
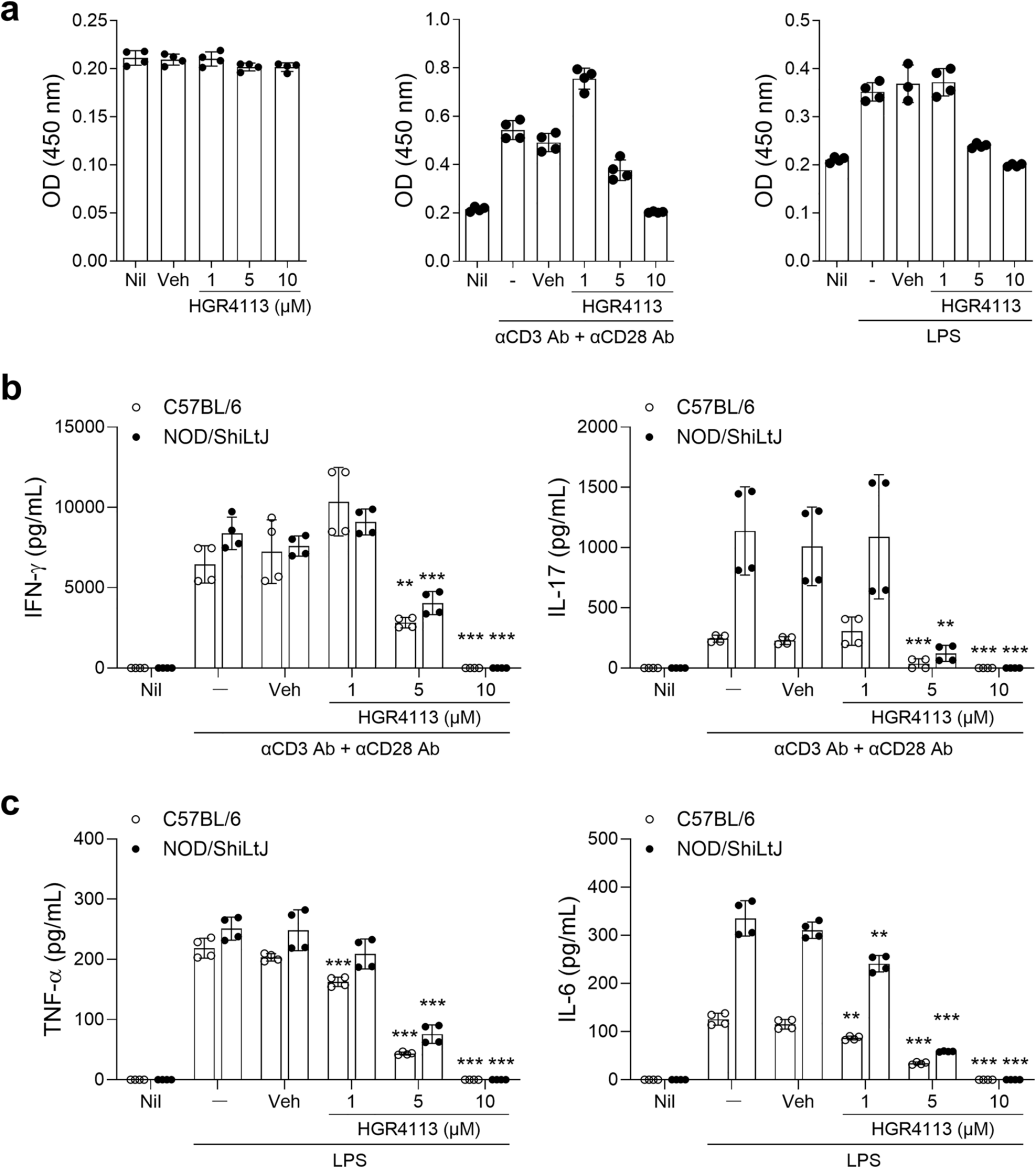
HGR4113 treatment decreases the production of inflammatory cytokines in vitro. **a** Splenocytes from C57BL/6 mice were stimulated with HGR4113 (1, 5 or 10 μM) or vehicle for 2 h and then cultured with or without anti-CD3 antibody (Ab) (0.5 μg/ml)/anti-CD28 Ab (1 μg/ml) or lipopolysaccharide (LPS; 100 ng/ml) for 3 days. After 3 days, splenocyte viability was determined by the CCK-8 assay. **b** Splenocytes from C57BL/6 or NOD/ShiLtJ mice were stimulated with HGR4113 (1, 5, or 10 μM) or vehicle for 2 h and then cultured with anti-CD3 Ab (0.5 μg/ml)/anti-CD28 Ab (1 μg/ml) for 3 days. IFNγ and IL-17 levels in culture supernatant were determined using ELISA. **c** Splenocytes from C57BL/6 or NOD/ShiLtJ mice were stimulated with HGR4113 (1, 5, or 10 μM) or vehicle for 2 h and then cultured with LPS (100 ng/ml) for 3 days. TNF and IL-6 levels in culture supernatant were determined using ELISA. Data are presented as the mean ± SD of three independent experiments. Statistical significance was determined by one-way ANOVA followed by Tukey’s honestly significant difference post hoc test. **P < 0.01, ***P < 0.001 versus vehicle-treated control.

To determine the effects of HGR4113 on cell proliferation in vitro, splenocytes from C57BL/6 mice were pretreated with HGR4113 for 2 h and then cultured with or without anti-CD3 antibody (Ab)/anti-CD28 Ab or LPS for 3 days. HGR4113 treatment did not affect the viability of splenocytes that were not stimulated with anti-CD3 Ab/anti-CD28 Ab or LPS. However, HGR4113 treatment dose-dependently decreased the viability of splenocytes stimulated with anti-CD3 Ab/anti-CD28 Ab or LPS (Fig. 1a). To assess the impact of HGR4113 on the activation of SS-derived pathogenic immune cells, splenocytes from NOD/ShiLtJ mice exhibiting SS-like symptoms were stimulated with HGR4113 in the presence of anti-CD3 Ab/anti-CD28 Ab or LPS for 3 days. HGR4113 treatment effectively reduced the amount of IFNγ that was increased by anti-CD3 Ab and anti-CD28 Ab stimulation. Compared with normal C57BL/6 mice, production of IL-17 after anti-CD3 Ab and anti-CD28 Ab stimulation in splenocytes of NOD/ShiLtJ mice was significantly increased, and HGR4113 significantly reduced IL-17 levels (Fig. 1b). Additionally, treatment with HGR4113 effectively reduced the amount of TNF that was increased by LPS stimulation. Compared with normal C57BL/6 mice, LPS stimulation significantly increased the production of IL-6 in splenocytes of NOD/ShiLtJ mice, and HGR4113 treatment significantly reduced IL-6 production (Fig. 1c). These results demonstrate that HGR4113 suppresses the production of inflammatory factors concomitant with reduced cellular viability under stimulatory conditions in immune cells in a preclinical model of SS.

### HGR4113 inhibits IL-17 production by regulating STAT3 activity and the metabolic profile in splenic CD4^+^ T cells

**Fig. 2:**
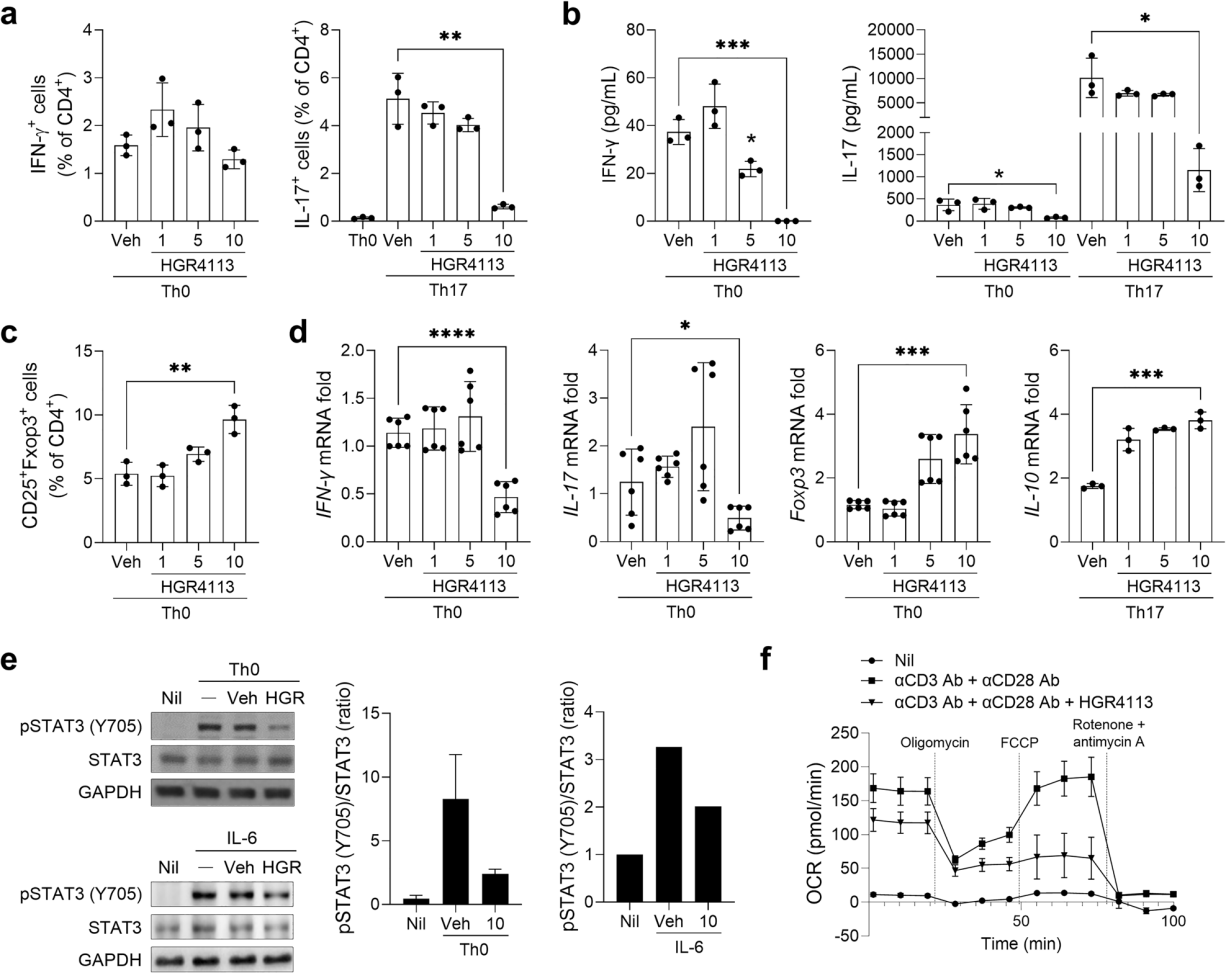
HGR4113 inhibits IL-17 production by suppressing STAT3 phosphorylation and altering the metabolic profile in splenic CD4^+^ T cells. **a–d** Splenic CD4^+^ T cells from C57BL/6 mice were stimulated with HGR4113 (1, 5 or 10 μM) or vehicle for 2 h and then cultured with anti-CD3 antibody (Ab) (0.5 μg/ml)/anti-CD28 Ab (1 μg/ml) or under Th17-polarizing conditions (anti-CD3 Ab, anti-CD28 Ab, anti-IFNγ Ab/anti-IL-4 Ab, each at 5 μg/ml; TGF-β, 2 μg/ml; IL-6, 20 ng/ml) for 3 days. The frequencies of CD4^+^IFNγ^+^ (Th1), CD4^+^IL-17^+^ (Th17) (**a**) and CD4^+^CD25^+^Foxp3^+^ (Treg) cells (**c**) were assessed by flow cytometry. IFNγ and IL-17 levels in culture supernatant were determined using ELISA (**b**). *IFNγ*, *IL-17*, *Foxp3* and *IL-10* mRNA expression was determined using real-time PCR (**d**). **e** Splenic CD4^+^ cells from C57BL/6 mice were stimulated with HGR4113 (10 μM) or vehicle for 2 h and then cultured with or without IL-6 (20 ng/ml) for 30 min or anti-CD3 Ab (0.5 μg/ml)/anti-CD28 Ab (1 μg/ml) for 12 h. The levels of p-STAT3 (Tyr705) and STAT3 were measured by immunoblotting. The graphs are representative of three independent experiments. **f** Splenic CD4^+^ T cells from C57BL/6 mice (*n* = 4) were stimulated with HGR4113 (10 μM) or vehicle for 2 h and then cultured with anti-CD3 Ab/anti-CD28 Ab for 12 h. OCR changes during treatment with oligomycin (10 μM), FCCP (3 μM), and rotenone plus antimycin A (10 μM) were assessed by extracellular flux analysis. Data are presented as the mean ± SD of three independent experiments. Statistical significance was determined by one-way ANOVA followed by Tukey’s honestly significant difference post hoc test. *P < 0.05, **P < 0.01, ***P < 0.001, ****P < 0.0001 versus vehicle-treated control.

To determine whether HGR4113 affects cytokine secretion in T cells, splenic CD4^+^ T cells isolated from C57BL/6 mice were treated with HGR4113 and cultured under anti-CD3 Ab and anti-CD28 Ab stimulation or under Th17-polarizing conditions for 3 days. HGR4113 dose-dependently suppressed the frequency of Th17 cells under Th17-polarizing conditions (Fig. 2a). Treatment with HGR4113 also dose-dependently suppressed the amounts of IFNγ and IL-17 in the culture supernatant of CD4^+^ T cells compared with untreated cells (Fig. 2b). Notably, despite the suppression of effector cytokines, HGR4113 dose-dependently enhanced differentiation into regulatory T cells (Tregs) under anti-CD3 Ab and anti-CD28 Ab stimulation (Fig. 2c). Treatment with HGR4113 downregulated *IFNγ* and *IL-17* mRNA expression under anti-CD3/anti-CD28 Ab stimulation and upregulated the levels of *Foxp3* and *IL-10* mRNA under anti-CD3/anti-CD28 Ab or Th17-polarizing conditions, respectively (Fig. 2d). Given that STAT3 is a key transcription factor in Th17 cells, we investigated whether HGR4113 regulates STAT3 activity. Splenic CD4^+^ T cells from C57BL/6 mice were pretreated with HGR4113 for 2 h and then cultured for 12.5 h under anti-CD3/anti-CD28 Ab or IL-6 stimulation; levels of phosphorylated STAT3 (Tyr705) were measured. HGR4113 inhibited phosphorylation of STAT3 induced by each stimulus (Fig. 2e). To assess the effects of HGR4113 on mitochondrial respiration, splenic CD4^+^ T cells were treated with HGR4113 and the OCR was measured. HGR4113 significantly reduced the increased oxidative phosphorylation (OXPHOS) activity induced by anti-CD3/anti-CD28 Ab stimulation (Fig. 2f). These results indicate that HGR4113 preferentially suppresses Th17 differentiation and OXPHOS in CD4^+^ T cells while promoting a regulatory phenotype.

### HGR4113 increases the frequency of IL-10-producing regulatory B cells and decreases immunoglobulin production

**Fig. 3:**
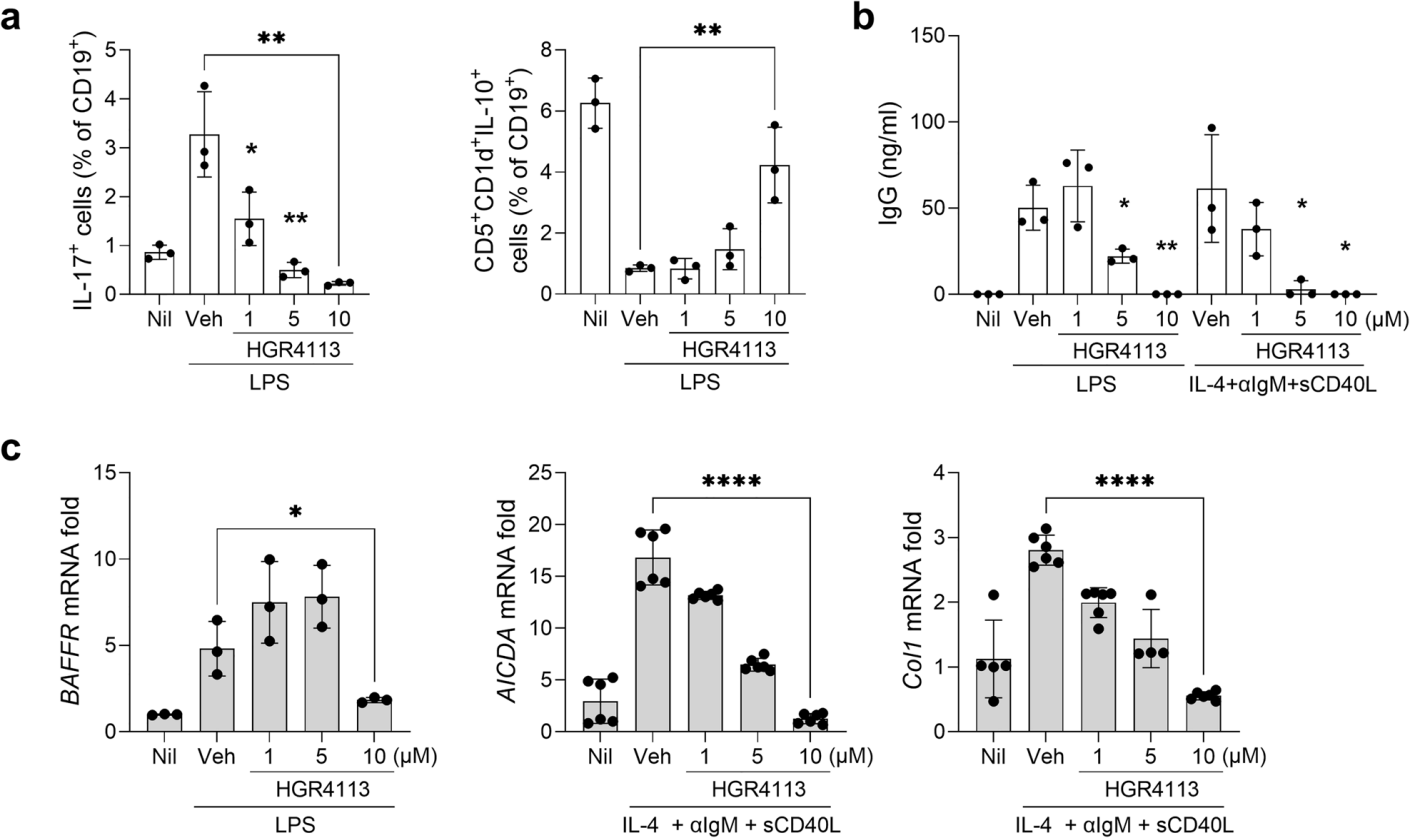
HGR4113 inhibits immunoglobulin production in splenic CD19^+^ B cells. **a–c** Splenic CD19^+^ B cells from C57BL/6 mice were stimulated with HGR4113 (1, 5 or 10 μM) or vehicle for 2 h and then cultured with LPS (100 ng/ml) or anti-IgM antibody (10 μg/ml), IL-4 (20 ng/ml), and soluble CD40L (1 μg/ml) for 4 days. The frequencies of CD19^+^IL-17^+^ cells and B10 cells (CD19^+^CD5^+^CD1d^+^IL-10^+^) were assessed by flow cytometry (**a**). Immunoglobulin levels in culture supernatant were determined using ELISA (**b**). *BAFF* receptor, *AICDA* and *Col1* mRNA expression was determined using real-time PCR (**c**). Data are presented as the mean ± SD of three independent experiments. Statistical significance was determined by one-way ANOVA followed by Tukey’s honestly significant difference post hoc test. LPS, lipopolysaccharide. *P < 0.05, **P < 0.01, ****P < 0.0001 versus vehicle-treated control.

To determine whether HGR4113 modulates B cell function, splenic CD19^+^ B cells isolated from C57BL/6 mice were treated with HGR4113 and cultured under LPS stimulation for 4 days. HGR4113 dose-dependently reduced the frequency of IL-17-producing B cells induced by LPS stimulation. In contrast, HGR4113 significantly increased the frequency of IL-10-producing regulatory B cells (B10 cells), which were otherwise reduced by LPS stimulation (Fig. 3a). HGR4113 treatment also dose-dependently reduced immunoglobulin production under LPS stimulation or stimulation that activates B cell receptors (Fig. 3b). Furthermore, HGR4113 downregulated the expression of *BAFFR* mRNA increased by LPS stimulation and attenuated the mRNA levels of *AICDA* and *collagen 1* increased by BCR activation (Fig. 3c). This reciprocal effect on inflammatory versus regulatory B cell subsets suggests that HGR4113 induces a functional shift in B cell populations rather than a non-specific suppression of B cell activity.

### HGR4113 attenuates the severity of Sjögren’s syndrome in NOD/ShiLtJ mice

**Fig. 4:**
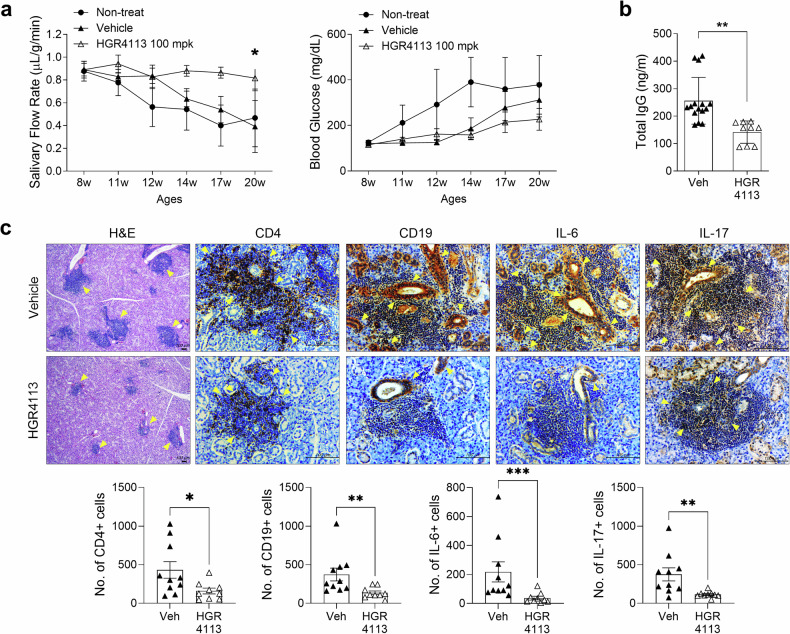
HGR4113 attenuates the severity of Sjögren’s syndrome in NOD/ShiLtJ mice. Eight-week-old female NOD/ShiLtJ mice were orally administered vehicle or HGR4113 (100 mg/kg) daily for 12 weeks. **a** Salivary flow rate (left) and blood glucose level (right) were measured at weeks 8, 11, 12, 14, 17 and 20 after HGR4113 administration (*n* = 10/group). **b** Total IgG in serum collected from 20-week-old mice was measured by ELISA (*n* = 15 for vehicle, *n* = 9 for HGR4113). **c** Salivary gland sections from 20-week-old mice were stained with haematoxylin and eosin (H&E), CD4, CD19, IL-6 and IL-17 (*n* = 10 for vehicle, n = 9 for HGR4113). Representative histological features are shown. Yellow arrowheads indicate representative areas of inflammatory-cell infiltration and positive staining for CD4, CD19, IL-6 and IL-17, respectively. The graph shows the histological grade. Data are presented as the mean ± SD of three independent experiments. Statistical significance was determined by one-way analysis of variance followed by Tukey’s honestly significant difference post hoc test. *P < 0.05, **P < 0.01, ***P < 0.001 versus vehicle-treated control.

To evaluate the therapeutic potential and systemic safety of HGR4113 in SS progression, 8-week-old female NOD/ShiLtJ mice were administered HGR4113 (100 mg/kg) or vehicle orally once daily for 12 weeks. With increasing age, the salivary flow rate decreased in the vehicle-treated group; this decrease was alleviated in the HGR4113-treated group (Fig. 4a).

Importantly, blood glucose (Fig. 4a) and body weights (Supplementary Fig. 1a) remained stable throughout the treatment period, suggesting that HGR4113 was well-tolerated without inducing overt systemic metabolic impairment. After 12 weeks of HGR4113 administration, serum IgG levels were significantly lower in HGR4113-treated mice than in the vehicle-treated group (Fig. 4b). In the HGR4113 group, salivary gland inflammation was reduced compared with controls (Fig. 4c). To assess the effect of HGR4113 on inflammatory cell infiltration into the salivary glands, salivary gland sections were immunohistochemically stained for CD4, CD19, IL-6 and IL-17. In the HGR4113 group, infiltration of cells expressing CD4 or CD19, as well as cells expressing the inflammatory cytokines IL-6 or IL-17, was notably attenuated in the salivary gland tissue (Fig. 4c). These results demonstrate that HGR4113 alleviates the development of SS-like symptoms in NOD/ShiLtJ mice while maintaining favourable systemic tolerability.

### HGR4113 decreases the frequency of ex vivo inflammatory cells and fibrosis in NOD/ShiLtJ mice

**Fig. 5:**
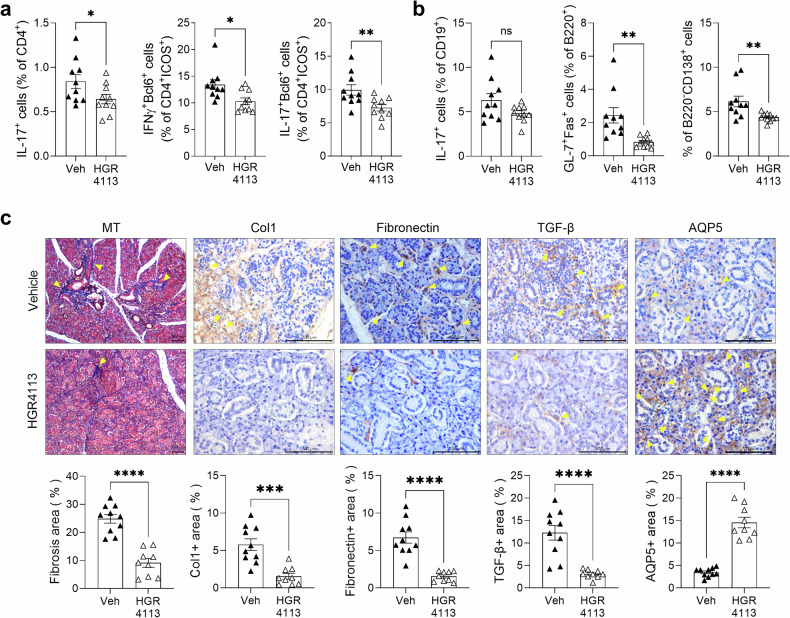
HGR4113 treatment decreases the frequency of ex vivo inflammatory cells and fibrosis in NOD/ShiLtJ mice. Eight-week-old female NOD/ShiLtJ mice were given vehicle or HGR4113 (100 mg/kg) orally daily for 12 weeks. **a**, **b** Ex vivo splenocytes from 20-week-old NOD/ShiLtJ mice were cultured with PMA, ionomycin and GolgiStop for 4 h and then immunostained with antibodies against CD4^+^IL-17^+^, CD4^+^ICOS^+^IFNγ^+^Bcl6^+^, CD4^+^ICOS^+^IL-17^+^Bcl6^+^ (**a**), CD19^+^IL-17^+^, B220^+^GL-7^+^Fas^+^, and B220^−^CD138^+^ cells (**b**) (*n* = 10/group). The cells were analysed by flow cytometry. **c** Salivary gland sections from 20-week-old mice were stained with Masson’s trichrome (MT), collagen I (Col1), fibronectin, TGFβ and aquaporin-5 (AQP5) (*n* = 10 for vehicle, *n* = 9 for HGR4113). Representative histological features are shown, and the graph displays the histological grade. Yellow arrowheads indicate representative areas of collagen deposition (MT), positive staining for fibrosis markers (Col1, Fibronectin and TGFβ), and restored expression of AQP5 in salivary-gland tissues. Data are presented as the mean ± SD. Statistical significance was determined by one-way analysis of variance followed by Tukey’s honestly significant difference post hoc test. *P < 0.05, **P < 0.01, ***P < 0.001, ****P < 0.0001 versus vehicle-treated control.

To determine how HGR4113 treatment affects pathogenic cells in NOD/ShiLtJ mice, we analysed the populations of T and B cell subsets in ex vivo splenocytes from HGR4113-treated NOD/ShiLtJ mice. HGR4113 treatment significantly reduced the frequencies of splenic Th17 cells and CD4^+^ICOS^+^IFNγ^+^Bcl6^+^ and CD4^+^ICOS^+^IL-17^+^Bcl6^+^ follicular helper T (Tfh) cells compared with the vehicle-treated group (Fig. 5a). Additionally, we assessed the frequency of CD4^+^ CD25^+^ Foxp3^+^ regulatory T (Treg) cells in the spleen. Although HGR4113 promoted Treg differentiation in vitro (Fig. 2c), the frequency of splenic Treg cells did not show a statistically significant difference between the vehicle and HGR4113-treated groups (data not shown). This suggests that in the complex in vivo environment, the therapeutic impact of HGR4113 is characterized primarily by the attenuation of pathogenic Th1, Th17 and Tfh effector populations rather than a systemic expansion of the Treg pool. HGR4113 treatment also significantly reduced the frequencies of splenic IL-17-producing B cells, germinal-centre B cells, and plasma cells compared with the vehicle-treated group (Fig. 5b). Increased fibrosis in the minor labial salivary glands of patients with primary SS correlates with the focus score and leads to impaired function^17,18^. To investigate the effect of HGR4113 on fibrosis development, the extent of fibrosis was examined by staining the salivary glands of HGR4113-treated mice with Masson’s trichrome. HGR4113 treatment reduced collagen deposition in the salivary glands (Fig. 5c). It also reduced the infiltration of cells positive for collagen type I and fibronectin-crucial components of the extracellular matrix-into salivary gland tissue compared with the vehicle-treated group (Fig. 5c). Furthermore, HGR4113 treatment reduced the infiltration of cells positive for TGF-β, which plays a pivotal role in the pathogenesis of fibrosis^19^, into salivary gland tissue (Fig. 5c). In contrast, HGR4113 treatment increased the infiltration of cells positive for aquaporin-5, a water channel predominantly located in the apical membrane of salivary-gland acinar cells^20^, into salivary-gland tissue (Fig. 5c). These results demonstrate that HGR4113 attenuates the systemic expansion of pathogenic immune cells and preserves the structural integrity of salivary glands by mitigating fibrosis in NOD/ShiLtJ mice.

### 3D salivary epithelial structure formation in HGR4113-treated NOD/ShiLtJ mice

**Fig. 6:**
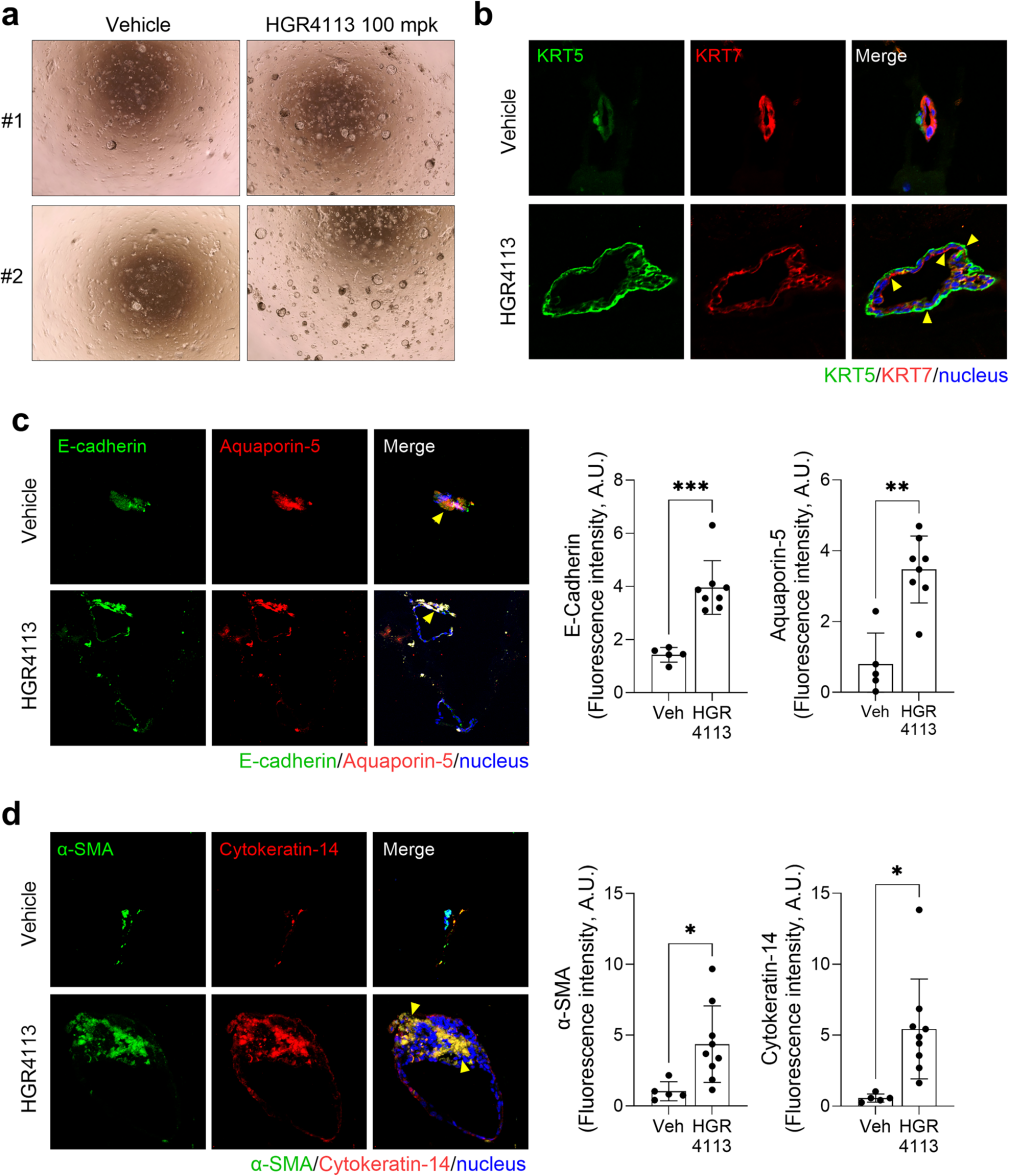
3D salivary epithelial structure formation in HGR4113-treated NOD/ShiLtJ mice. Eight-week-old female NOD/ShiLtJ mice were orally administered vehicle or HGR4113 (100 mg/kg) daily for 12 weeks. Salivary epithelial cells isolated from the submandibular glands of 20-week-old NOD/ShiLtJ mice were cultured for 10 days. For quantification, *n* = 5 representative samples (based on mean salivary flow rate) for the vehicle group and *n* = 8–9 for the HGR4113 group were used. The variation in *n* in the HGR4113 group was due to sample exhaustion during multiple rounds of immunofluorescence staining. **a** Bright-field images of 3D salivary epithelial cultures. **b** Representative immunofluorescence images of basal cells (KRT5; green) and luminal cells (KRT7; red) in 3D salivary epithelial cultures. **c** Representative immunofluorescence images of epithelial cell adherens junctions (E-cadherin; green) and acinar cells (AQP5; red) in 3D salivary epithelial cultures. **d** Representative immunofluorescence images of myoepithelial cells (α-SMA; green; cytokeratin-14; red) in 3D salivary epithelial cultures. Yellow arrowheads indicate representative regions of structural maturation and lineage-specific marker expression (KRT5, KRT7, E-cadherin, AQP5, α-SMA and KRT14) within the 3D salivary epithelial cultures. Data are presented as the mean ± SD. Statistical significance was determined by one-way ANOVA followed by Tukey’s honestly significant difference post hoc test. *P < 0.05, **P < 0.01, ***P < 0.001 versus vehicle-treated control. The English in this document has been checked by at least two professional editors, both native speakers of English. For a certificate, please see: http://www.textcheck.com/certificate/o3xXlY.

Considering that HGR4113 improved fibrosis in salivary gland tissue and increased aquaporin-5 expression (Fig. 5c), we isolated salivary epithelial cells from the salivary gland tissue of NOD/ShiLtJ mice administered HGR4113 to determine whether the treatment enhanced the reparative capacity of these cells. After 10 days of culture, we observed substantial acceleration of 3D epithelial structure development from salivary epithelial cells of NOD/ShiLtJ mice given HGR4113 compared with those given vehicle (Fig. 6a). 3D salivary epithelial cultures should contain secretory acinar cells and surrounding myoepithelial cells, which play essential roles in proper saliva secretion and overall gland function^21,22^. Compared with controls, 3D structures formed from salivary epithelial cells of NOD/ShiLtJ mice administered HGR4113 retained KRT5^+^ basal and KRT7^+^ luminal duct cells (Fig. 6b). Moreover, compared with the control, 3D salivary epithelial structures derived from salivary epithelial cells of NOD/ShiLtJ mice administered HGR4113 expressed aquaporin-5 and E-cadherin more strongly, markers of acinar cells and epithelial cell adherens junctions, respectively (Fig. 6c). 3D salivary epithelial cultures developed from salivary epithelial cells of NOD/ShiLtJ mice administered HGR4113 also contained KRT14^+^αSMA^+^ myoepithelial cells (Fig. 6d). These results demonstrate that HGR4113 promotes the structural and functional maturation of 3D salivary epithelial structures, suggesting that HGR4113-mediated environment improves the inherent reparative capacity of the salivary epithelium in SS-like NOD/ShiLtJ mice.

## Discussion

In this study, we evaluated the effect of the synthetic glabridin derivative HGR4113 on modulating the severity of SS in SS-like NOD/ShiLtJ mice. HGR4113 reduced the production of inflammatory cytokines induced by TCR or LPS stimulation in splenocytes derived from NOD/ShiLtJ mice. In particular, it reduced the development of Th17 cells by downregulating STAT3 phosphorylation in CD4^+^ T cells and promoted the development of IL-10-producing regulatory B cells in CD19^+^ B cells. HGR4113 administration effectively alleviated the development of SS in NOD/ShiLtJ mice by reducing the frequency of inflammatory cells in the spleen, alleviating fibrosis and increasing AQP5 expression in salivary gland tissue. HGR4113 treatment also promoted salivary gland 3D salivary epithelial structure formation in vitro and in vivo.

HGR4113 is a synthetic glabridin derivative with a similar skeletal structure; however, it differs substantially from glabridin in that it has a hydroxy-to-propoxy modification at the resorcinol ring at C-4 and double-bond hydrogenation in the pyranobenzene structure^23^. HGR4113 attenuates the LPS-mediated increase in mRNA levels of proinflammatory cytokines, NF-κB activation, and JNK phosphorylation in LPS-stimulated RAW264.7 macrophages. These structural differences result in improved chemical stability and metabolic characteristics compared with glabridin. Moreover, in LPS-stimulated RAW264.7 macrophages, HGR4113 downregulated LPS-mediated increases in mRNA levels of inflammatory cytokines, as well as NF-κB activation and JNK phosphorylation, similar to or more effectively than glabridin^23^. Although HGR4113 is currently in the data analysis phase of phase I clinical trials (NCT05642377) for type 2 diabetes mellitus, there are very few reports regarding its mechanism of action and efficacy. Although specific safety and tolerability data from this ongoing trial have not yet been publicly disclosed, the in vivo dose of 100 mg/kg was selected based on the Human Equivalent Dose conversion from the clinically relevant dose range (300–600 mg) explored in the aforementioned phase 1 trials (NCT05642377). Our preliminary dose-finding study comparing 100 mg/kg and 200 mg/kg, both groups showed similar initial improvements in saliva production at week 14, but the 100-mg/kg group maintained a consistent and significant therapeutic effect throughout the 20-week study period (Supplementary Fig. 2). In contrast, the 200-mg/kg group showed a decline in efficacy in the later phase (weeks 17–20), with saliva production returning to levels similar to those of the control group. This suggests that 100 mg/kg is the optimal therapeutic dose for HGR4113 in this chronic model, whereas excessively high doses may trigger compensatory feedback mechanisms or lead to potential systemic burden that may impair long-term recovery capacity. Furthermore, pharmacokinetic data indicate that oral administration of HGR4113 at 160 mg/kg in rodents achieves a plasma *C*_max_ of approximately 4.90 μg/ml^14^. When considering the molecular weight of HGR4113 (368.47 g/mol), this plasma exposure effectively covers the concentration range identified in our in vitro assay (1–10 μM). Importantly, the selected 100 mg/kg dose demonstrated excellent systemic safety, as demonstrated by the absence of significant changes in body weight or organ toxicity (Supplementary Fig. 1). Therefore, 100 mg/kg was confirmed as the most stable and efficacious dose for the long-term potential of HGR4113 in restoring salivary gland function.

Metabolic abnormalities have been reported to be closely related to the pathological development and exacerbation of SS. We have shown that butyric acid or *Lactobacillus rhamnosus*, which produces butyric acid, can directly increase salivary secretion and suppress autoimmune B cells and autoantibody production^24^. Serum leptin and IL-1β levels were elevated in SS patients with metabolic disorders, and disease severity was aggravated when a glucosamine–nitrosourea compound targeting pancreatic β cells was administered to NOD/ShiLtJ mice, a preclinical model of SS^25,26^. Relative to healthy individuals, patients with SS have altered mitochondrial DNA copy numbers and increased levels of factors associated with mitochondrial fission and transcription^27^. Furthermore, mitochondrial swelling and cristae disruption, as well as changes in mitochondria-related metabolic pathways, have been observed in immune cells infiltrating the salivary glands of SS patients^28^. These findings suggest that mitochondrial dysfunction is a key factor in the pathophysiology of SS and that mitochondria-targeting therapies may represent a novel treatment strategy.

In this study, we evaluated the therapeutic potential of HGR4113 in SS associated with metabolic abnormalities^8,9^. Phenotypic and functional changes observed during the proliferation and differentiation of B and T cells depend on dynamic mitochondrial metabolism. Resting B cells have low metabolic activity, whereas activation of B cells with BCR or LPS stimulation increases glucose uptake and glycolysis^29,30^. When T cells are activated through antigen recognition, they switch to a state of elevated OXPHOS and aerobic glycolysis to meet the high energy and biosynthetic demands required for rapid proliferation and effector activity^31,32^. Proinflammatory cytokines such as IL-6 and TNF activate glycolysis and anabolic processes to promote differentiation of effector T cells, including Th1, Th2 and Th17 cells^33^, whereas anti-inflammatory cytokines such as TGF-β promote oxidative metabolism and mitochondrial respiration to support the development of Tregs^34^, indicating that each T cell type has unique metabolic characteristics. HGR4113 effectively reduced the production of inflammatory cytokines and OXPHOS activity in CD4^+^ T cells by inhibiting STAT3 activity. Furthermore, HGR4113 reduced IgG production in CD19^+^ B cells and promoted their differentiation into regulatory B cells. These results demonstrate that HGR4113 not only alleviates inflammation but also modulates mitochondrial OXPHOS.

Research aimed at developing and utilizing salivary gland organoids is gaining increasing attention. A recent study demonstrated that salivary-gland organoids derived from patients with SS consistently activated IFN-related genes and that IFN signalling was reduced by treatment with a JAK inhibitor, suggesting that salivary-gland organoids derived from SS patients can recapitulate pathological characteristics of the disease^35^. Inhibition of activin receptor-like kinase (Alk) signalling during salivary-gland organoid generation has been shown to promote organoid production^36^. Furthermore, in radiation-induced salivary-gland damage, YAP translocated to the nucleus of salivary-gland cells and activated regeneration-related genes, thereby inducing the proliferation of ductal cells and promoting salivary-gland regeneration^37^. In this study, HGR4113 treatment significantly enhanced the formation and functional maturation of 3D salivary-gland epithelial structures. We evaluated the efficacy of HGR4113 in 3D salivary epithelial cultures from NOD/ShiLtJ mice. 3D salivary epithelial cultures derived from NOD/ShiLtJ mice administered HGR4113 exhibited enhanced structural development, with increased development of KRT5^+^ basal and KRT7^+^ luminal duct cells, aquaporin-5^+^ acinar cells and KRT14^+^αSMA^+^ myoepithelial cells. However, because this study model focused on immediate reparative responses rather than long-term viability, it is important to distinguish these results from true regeneration, which requires long-term self-renewal over multiple passages (e.g. 3–4 passages or more). Furthermore, this improvement in 3D structure formation may be a consequence of a more favourable tissue environment due to HGR4113-induced suppression of inflammation and fibrosis. Nevertheless, the significant increase in Ki-67 expression in the HGR4113-treated group (Supplementary Fig. 3) suggests that this treatment does not simply induce rapid differentiation at the expense of environmental changes or stem-cell properties but rather induces a robust proliferative response in the epithelial progenitor-cell population. Collectively, these results suggest that HGR4113 promotes functional restoration of the salivary gland by stabilizing the tissue microenvironment and stimulating the inherent reparative capacity of the epithelial-cell population. Further studies utilizing lineage tracing and long-term culture protocols are needed to fully elucidate the long-term regenerative potential.

In SS, B cell-targeted therapies such as rituximab are effective in some patients, but they fail to completely address T cell-mediated immune responses. HGR4113 exhibits potent inhibitory effects on both pathogenic T and B cells by metabolic intervention. Furthermore, although BAFF inhibitors such as belimumab primarily focus on B cell survival, HGR4113 inhibits BAFF receptor expression and directly suppresses inflammatory cytokine (IFNγ, IL-17) production. Although JAK/STAT inhibitors exhibit broad systemic immunosuppressive effects, HGR4113 preferentially impacts STAT3 phosphorylation and mitochondrial stability, potentially offering a more targeted metabolic intervention with superior stability in hyper-activated immune cells. Despite these promising results, this study has several limitations. First, although HGR4113 promoted Treg differentiation and Foxp3 expression in vitro, we did not observe a significant increase in splenic Treg populations ex vivo (data not shown). This suggests that in vivo efficacy may depend more on direct suppression of effector cells than on systemic Treg proliferation. Second, although HGR4113 has been identified as a potential treatment for SS, the precise molecular targets regulating STAT3 phosphorylation and mitochondrial metabolism remain to be fully elucidated. Third, our 3D salivary epithelial culture experiments involved asymmetric sample sizes (*n* = 5 for vehicle versus *n* = 8–9 for HGR4113). Although the attrition was based on documented experimental criteria, the pre-selection of vehicle subjects using mean salivary flow rate may introduce a potential selection bias. This strategy was employed to ensure a stable baseline for functional assessment, yet it necessitates cautious interpretation regarding the generalizability of the results. Finally, although HGR4113 is currently in the data analysis phase (active, not recruiting) of a phase 1 clinical trial for type 2 diabetes (NCT05642377), its specific safety and efficacy profile in SS patients has not yet been established. Although the trial is in its final stages, specific safety and tolerability data have not yet been publicly disclosed, which remains a limitation in fully correlating our preclinical findings with clinical safety. Even though the dose-dependent reduction in cell viability under stimulatory conditions (Fig. 1a) indicates an inhibitory effect on hyper-activated cells, the concurrent promotion of Treg and B10 cells suggests that HGR4113 does not induce generalized immune suppression. Rather, it appears to recalibrate the immune threshold by selectively curtailing the metabolic fitness of pathogenic effector subsets.

This study revealed that in a preclinical mouse model of SS, HGR4113 preferentially suppressed inflammation involving T and B cells, modulated mitochondrial metabolism, and promoted the formation and functional maturation of 3D salivary epithelial structures. These findings demonstrate the potential for developing metabolism-based disease-modifying therapeutics tailored to the pathophysiology of SS.

## Supplementary information

Supplementary Information
