# Supplementary material for: Therapeutic effects of a synthetic glabridin derivative on metabolic immuno-regulation and salivary gland functional restoration in experimental Sjögren’s syndrome
Source: Exp Mol Med. 2026 Jul 21;58(7):2356–67. doi: 10.1038/s12276-026-01778-0 (PMC13434468; doi:10.1038/s12276-026-01778-0)
Supplement: Supplementary file 1 [file 12276_2026_1778_MOESM1_ESM.pdf]

Supplementary Fig. 1

a

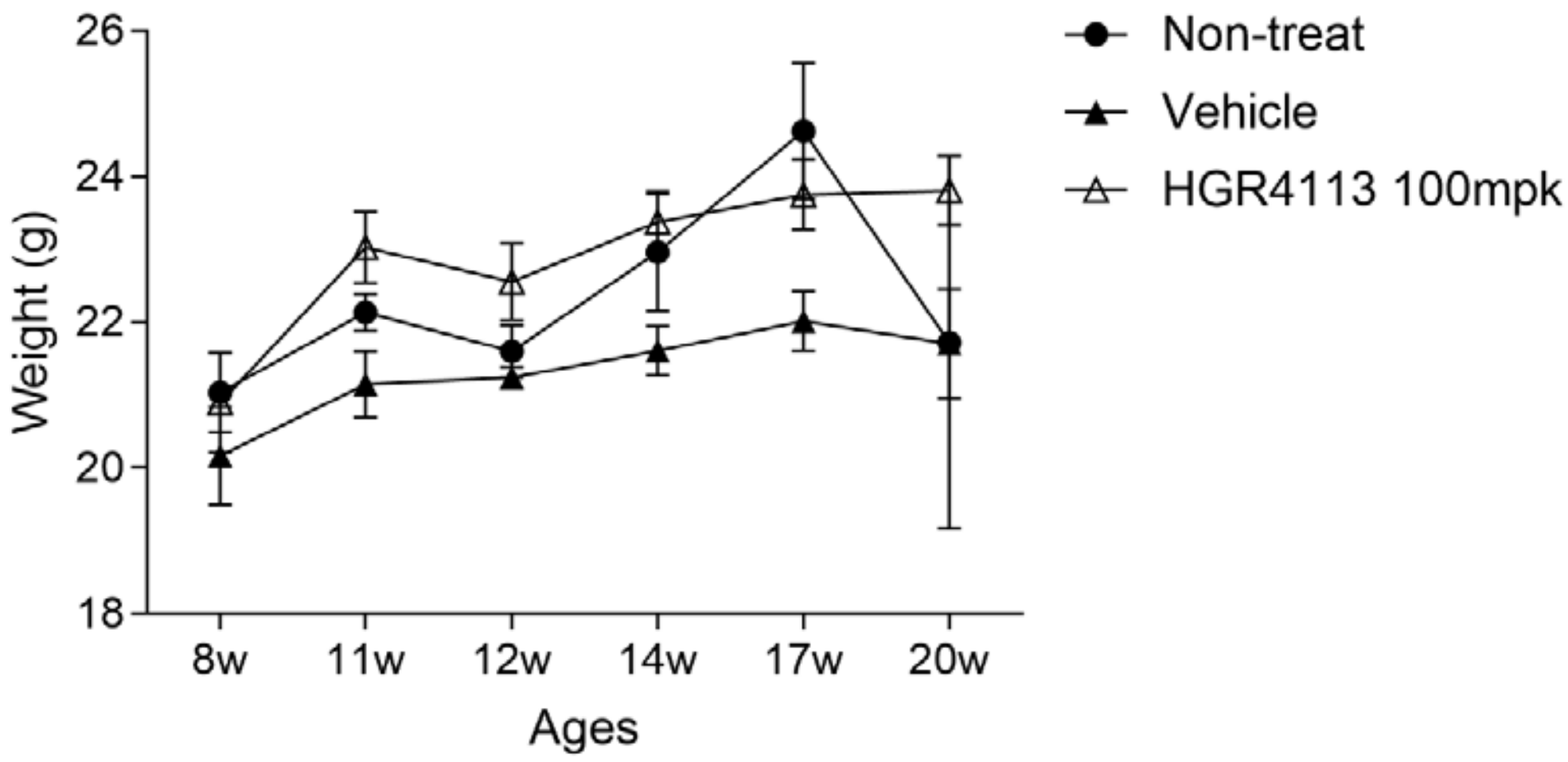

b

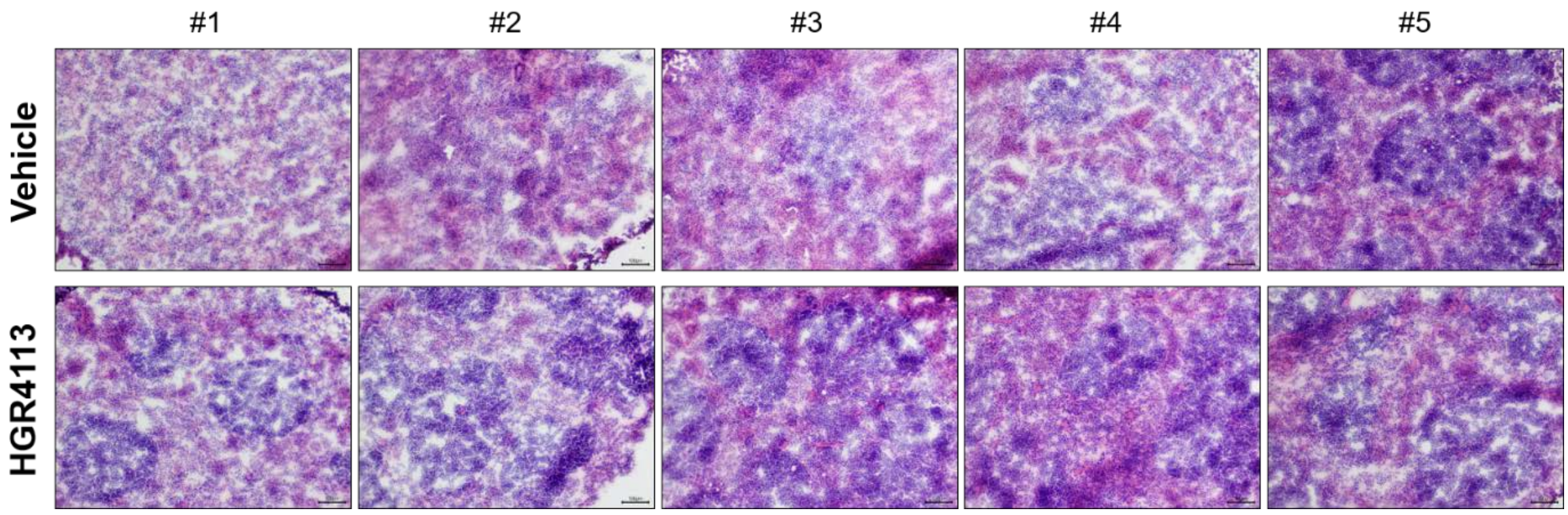

Supplementary Fig. 1: Safety assessment of long-term HGR4113 administration. Eight-week-old female NOD/ShiLtJ mice were given vehicle or HGR4113 (100 mg/kg) orally daily for 12 weeks. **a** Body weight changes in non-treated, vehicle, and HGR4113-treated groups (n = 10/group). No significant differences in body weight gain were observed among the groups. **b** Representative H&E staining of the spleen from vehicle- and HGR4113-treated (100 mg/kg) NOD/ShiLtJ mice from at 20 weeks of age (n = 5/group). At 100x magnification, the HGR4113-treated group exhibited more distinct and densely packed white pulp (WP) regions with well-defined boundaries compared to the vehicle group. No evidence of lymphoid depletion, follicular atrophy, or structural disruption was observed in the HGR4113-treated group, confirming the systemic safety of the chronic dosing regimen. Original magnification, 100x. (Scale bar = 100 μm).

Supplementary Fig. 2

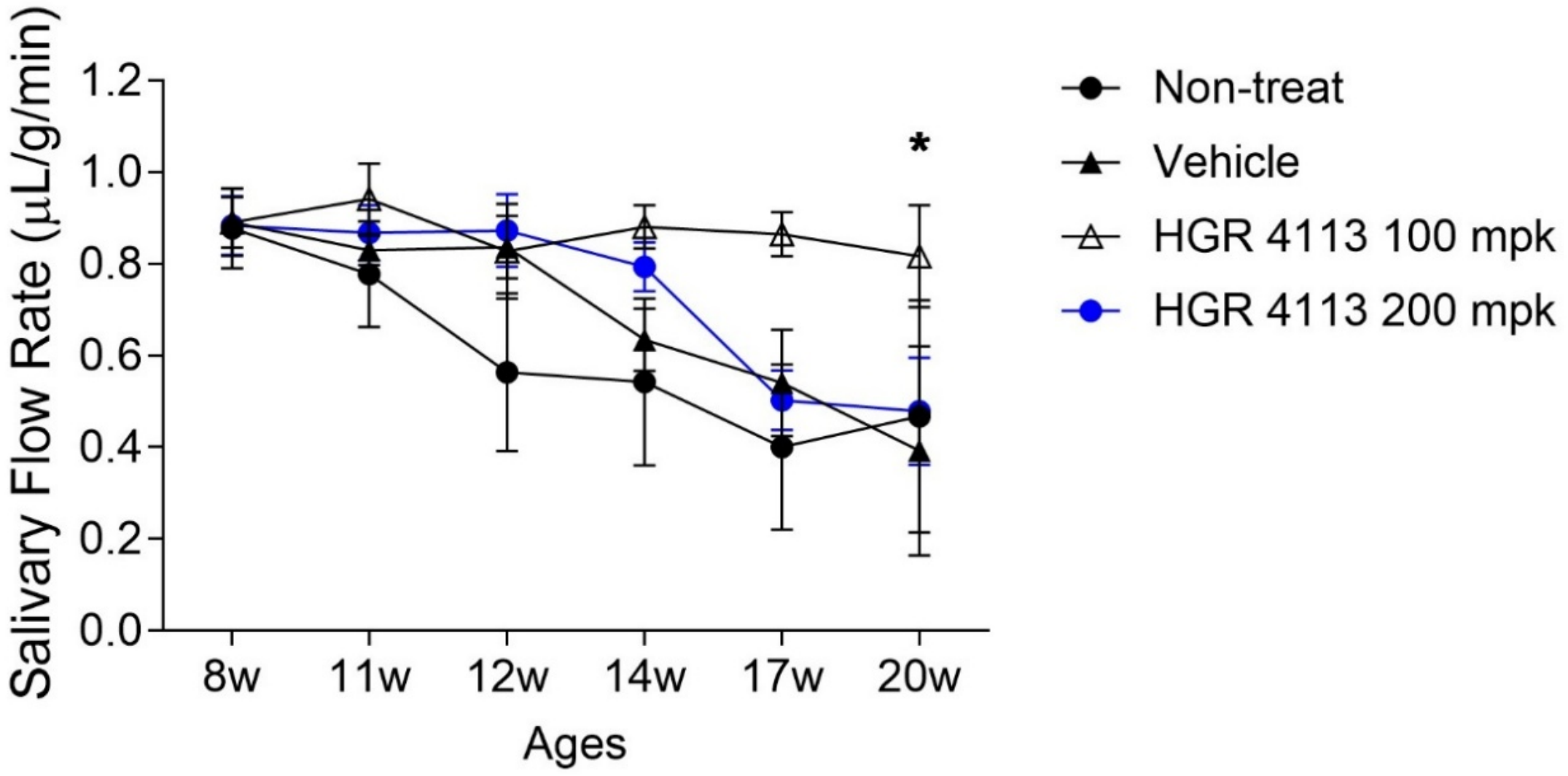

Supplementary Fig. 2: Dose-dependent effects of HGR4113 on salivary gland function.

Eight-week-old female NOD/ShiLtJ mice were given vehicle or HGR4113 (100 or 200 mg/kg) orally daily for 12 weeks. Salivary flow rate was measured at weeks 8, 11, 12, 14, 17, and 20 after HGR4113 administration (n = 10/group). While both HGR4113-treated groups showed initial functional improvement, the 100 mg/kg group exhibited a sustained and significant therapeutic effect through week 20, whereas the 200 mg/kg group showed a decline in efficacy in the later phase. Data represent mean ± SD. *$P < 0.05$ vs. Vehicle-treated control at 20 weeks.

Supplementary Fig. 3

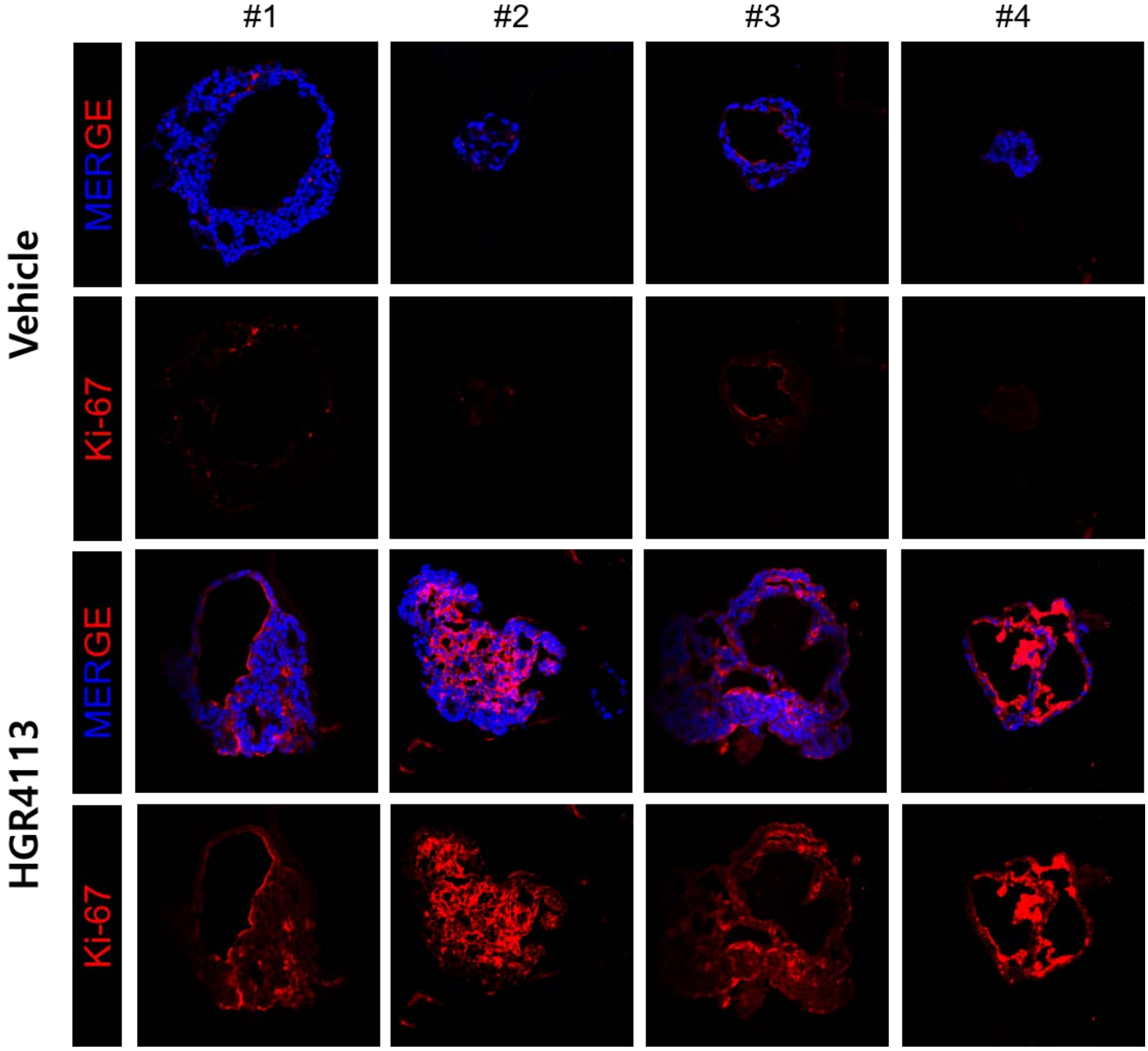

Supplementary Fig. 3: HGR4113 induces active proliferation in 3D salivary epithelial cultures.

Eight-week-old female NOD/ShiLtJ mice were given vehicle or HGR4113 (100 mg/kg) orally daily for 12 weeks. Salivary epithelial cells isolated from the submandibular glands of 20-week-old NOD/ShiLtJ mice were cultured for 10 days. Representative immunofluorescence images of Ki-67 (red) and DAPI (blue, MERGE) in 3D salivary epithelial cultures treated with vehicle or HGR4113. Four independent representative areas (#1–#4) are shown for each group. HGR4113-treated cultures exhibited a marked increase in Ki-67-positive cells compared to the vehicle group, indicating that the observed structural restoration is driven by an active proliferative response of the epithelial progenitor cell population. Original magnification, 200 x
